# Complete heart block is a significant predictor of mortality in immune checkpoint inhibitor myocarditis

**DOI:** 10.1186/s40959-023-00185-y

**Published:** 2023-09-20

**Authors:** Michael P. O’Shea, Suganya Arunachalam Karikalan, Ali Yusuf, Timothy Barry, Eiad Habib, John O’Shea, Michael Killian, Eman Baqal, Srishti Nayak, Rajeev Masson, Joerg Hermann, Shimoli Shah, Chadi Ayoub, Hicham El Masry

**Affiliations:** 1 Department of Cardiovascular Medicine, Mayo Clinic, 5777 E Mayo Boulevard, Phoenix, AZ 85054 USA; 2 Saint James’s Hospital, Dublin, Ireland; 3 Mayo Clinic Rochester, Rochester, MN USA

**Keywords:** Immune checkpoint inhibitors, Complete heart block, Myocarditis, Pharmacoepidemiology, Arrhythmias

## Abstract

**Background:**

Immune checkpoint inhibitor (ICI) myocarditis is associated with significant mortality risk. Electrocardiogram (ECG) changes in ICI myocarditis have strong prognostic value. However the impact of complete heart block (CHB) is not well defined. This study sought to evaluate the impact of CHB on mortality in ICI myocarditis, and to identify clinical predictors of mortality and CHB incidence.

**Methods:**

We conducted a retrospective cohort study of patients with ICI myocarditis at three Mayo Clinic sites from 1^st^ January 2010 to 31^st^ September 2022 to evaluate mortality rates at 180 days. Clinical, laboratory, ECG, echocardiographic, and cardiac magnetic resonance imaging (CMR) characteristics were assessed. Cox and logistic regression were performed for associations with mortality and CHB respectively.

**Results:**

Of 34 identified cases of ICI myocarditis, 7 (20.6%) had CHB. CHB was associated with higher mortality (HR 7.41, *p* = 0.03, attributable fraction 86.5%). Among those with CHB, troponin T (TnT) < 1000 ng/dL, low white blood cell count and high ventricular rate at admission were protective. There was trend towards increased survival among patients who underwent permanent pacemaker insertion (*p* = 0.051), although most experienced device lead complications. Factors associated with development of CHB included prolonged PR and QRS intervals and low Sokolow Lyon Index. Where these were normal and TnT was < 1000 ng/dL, no deaths occurred. Impaired myocardial longitudinal strain was sensitive for ICI myocarditis but was not prognostically significant.

**Conclusion:**

There is a strong temporal association between CHB and early mortality in people with ICI myocarditis. Focusing on arrhythmogenic complications can be helpful in predicting outcomes for this group of critically ill individuals.

## Introduction

Immune Checkpoint Inhibitor (ICI) therapy for cancer has increased substantially in the last decade with its ability to improve prognosis in a number of cancers [1]. ICI Myocarditis is a rare form of drug-induced myocardial injury characterized by infiltration of myocardial and skeletal muscle tissue with CD4 + and CD8 + T-cells [2]. Although increasingly recognized, the true incidence among those receiving ICI therapy is unclear [2, 3]. However, it is generally accepted to occur in < 1% of ICI therapy recipients. Mortality risk is high and has been estimated at 27% [2, 4].

A broad constellation of clinical data, biomarkers, electrocardiogram (ECG) and imaging parameters are used for diagnosis of myocarditis, and in some cases myocardial biopsy is undertaken [5]. ECG abnormalities in ICI myocarditis may include ventricular tachycardia and high-degree atrioventricular block, with conduction abnormalities carrying significant prognostic importance [4, 6, 7]. Other important characteristics include development of prolonged QRS, decrease in Sokolow-Lyon index, and development of pathologic Q waves [7]. The higher rates of arrhythmogenic fatality and conduction disorders are in keeping with histopathological involvement in the sinoatrial and atrioventricular nodes [2, 7]. Imaging characteristics of ICI myocarditis may differ from those in idiopathic or viral myocarditis. Cardiac magnetic resonance (CMR) has a high false negative value for ICI myocarditis compared to other types of acute myocarditis [4, 8, 9].

In the setting of suggested high mortality, and the need for a clear framework to guide clinical decision making, this study sought to examine the association between complete heart block (CHB) and mortality among patients with ICI myocarditis. Clinical demographics, biochemical and imaging characteristics were assessed for association with mortality rates and development of CHB among those with ICI myocarditis.

## Methods

### Study population

This study was approved by the Mayo Clinic Institutional Review Board (Application # 22–009077). Patients with ICI myocarditis were identified retrospectively from medical records using the Mayo Clinic Data Explorer system across all three Mayo Clinic Sites (Arizona, Florida and Minnesota). Patients who received ICI therapy and subsequently were diagnosed with myocarditis were identified between January 1^st^ 2010 and September 30^th^ 2023. Three Physician independent review with majority consensus was conducted to determine if a case met the criteria for possible, probable or definite myocarditis as defined by Bonaca et al., [5]. Patients who didn’t meet these criteria, or who had myocarditis prior to receiving ICI therapy, were excluded.

### Definitions

Case definition was the presence of acute ICI myocarditis, with associated complete heart block (CHB) during inpatient hospitalization on ECG or cardiac telemetry [5]. Controls were patients with ICI myocarditis who did not have CHB during hospitalization. The primary outcome was the mortality rate within 180 days of first administration of ICI. Secondary outcome was the development of CHB.

### Clinical characteristics

Clinical data were collected through review of electronic medical records. These included demographic information (age and sex), as well as pertinent co-morbidities (diabetes, hypertension, coronary artery disease, chronic obstructive lung disease, obstructive sleep apnea, prior stroke or transient ischemic attack, prior smoking, family history of premature coronary artery disease, hyperlipidemia, pericarditis, heart failure, acute coronary syndrome, prior conduction issues, prior atrial fibrillation, cancer type). Medications at admission were recorded, including anticoagulant, antihypertensive, statin, and antiplatelet agent use. Baseline and admission laboratory data were collected, including white blood cell count, 5^th^ generation Troponin T, creatine kinase (CK), aspartate aminotransferase (AST), alanine aminotransferase (ALT), albumin and N-terminal pro-brain natriuretic peptide (NT-Pro BNP).

Baseline and admission ECG data were collected. Sokolow Lyon Index (SLI) and T-wave inversion were calculated by single physician review (SAK, YA, MOS), while other measurements were based on machine calculated readings (ventricular rate, PR interval, QRS duration, QT interval, QTc interval, P/QRS/T axis). Data for transthoracic echocardiography (TTE) was collected, where available, prior to and after ICI myocarditis, and available CMR imaging reports were reviewed. Left ventricular longitudinal strain analysis was conducted retrospectively by a single operator (TB) on admission/post-admission TTEs using the same Echo Insight software for all patients. Global, apical, mid and basal strain were assessed. For key data-points relating to ICI diagnosis (biomarkers, CMR, TTE, and presence of CHB), independent dual-entry of data was performed to ensure accurate data entry.

### Analysis

Statistical analysis was performed using StataIC 16. Univariate cox regression was used to calculate and compare mortality rates among all people with ICI myocarditis. Attributable fraction was calculated using mortality rates. Nelson Aalen graphs were examined to assess validity of the proportional hazard assumption. Bivariate logistic regression was used to identify variables associated with development of CHB. Where significant data sparsity existed, Fisher’s exact test was used to assess for association between diagnostic testing and mortality risk at 180 days. Sensitivity was calculated for diagnostic testing. Associations with mortality risk and survival time among people with CHB were quantified using nonparametric methods, with the exception of use of linear regression and Pearson’s correlation coefficient where linear trend was identified on two-way scatter plot. Qualitative review was conducted among participants with ICI myocarditis with associated complete heart block (CHB).

## Results

A total of 51 charts were identified on initial screening, from 8522 patients who received at least one ICI during the study period. Following three-physician independent review, 34 patients met inclusion criteria for ICI myocarditis. Of these, 7 had complete heart block (CHB). Schoenfeld residual test demonstrated no evidence of violation of the proportional hazard assumption (χ^2^ = 0, *p* = 0.95). Mortality rate for all patients with ICI myocarditis in the 180 days following ICI administration was 0.176 deaths per 100 person-days (95% confidence interval [CI]: 0.091 to 0.338). Incidence risk for ICI myocarditis was 3.99 per 1000 patients treated. Incidence risk for ICI myocarditis with CHB was 0.82 per 1000 patients treated.

### Mortality with CHB

**Fig. 1:**
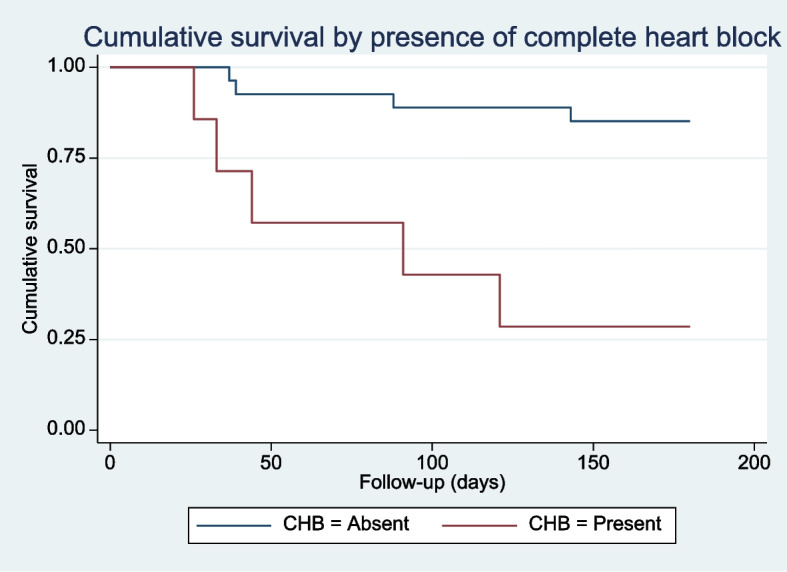
Kaplan Meyer Curve demonstrating cumulative survival among patients with ICI myocarditis, stratified by presence of CHB

Patients with CHB had substantially higher short-term mortality compared to those without CHB. Mortality rate among those with CHB was 0.74 deaths per 100 person-days (95% CI 0.31 to 1.78), while it was 0.09 (95% CI 0.03 to 0.24) for those without CHB (Fig. 1). Hazard ratio for presence of CHB was 7.41 (95% CI 1.96 to 28.04, *p* = 0.03, retrospective power ≅ 100%) [10]. The population attributable fraction of CHB to overall mortality rates in people with ICI myocarditis at 180 days was 86.5%. Nonparametric testing verified a strong association between CHB and mortality risk (Fisher’s exact *p* = 0.007) and survival time (Rank sum *p* = 0.0023) at 180 days.

### Characteristics of patients with CHB

All 7 patients who had CHB associated with ICI myocarditis had metastatic disease. Median survival 180 days after ICI administration was 91 days (Tables 1 and 2). All patients had elevated 5^th^ generation TnT, CK, WBC count and transaminases (AST and ALT). Shortness of breath, low TnT and WBC count and high HR were associated with reduced survival time (Tables 2 and 3), while ECG characteristic were otherwise not prognostic.

**Table 1:** Characteristics of people with immune checkpoint inhibitor myocarditis with associated complete heart block

**Age**	**Immune checkpoint inhibitor**	**Admission labs**		**Admission ECG**			**Treatment**					**Timeline** Time (days) from first ICI to		
		**TnT**	**WBC**	**QRS (msec)**	**PR (msec)**	**SLI (mV)**	**IVIG**	**PLEX**	**TVP**	**Permanent device**	Device lead complication*	**Myocarditis**	**CHB**	**Death**
83	Pembrolizumab	3409	19.2	136	**	12	-	-	-	-	-	24	24	26
79	Ipilimumab and Nivolumab	277	9.6	138	220	12	-	-	+	Leadless PPM	-	27	27	> 180
80	Nivolumab	620	6.7	134	182	11	-	-	+	Dual chamber PPM	-	28	28	121
79	Ipilimumab and Nivolumab	1162	13.9	124	166	5	+	-	+	-	-	25	26	33
58	Nivolumab	1169	10.3	130	204	3	+	-	+	Dual chamber PPM	A – lead dislodgement	22	22	44
70	Pembrolizumab	***	8.6	156	188	-	+	+	+	Dual chamber PPM	V—Failure to capture	19	21	> 180
81	Pembrolizumab	846	18	142	266	2	+	-	+	Dual chamber PPM	A – under-sensing	33	33	91

*ICI* immune checkpoint inhibitor, *IVIG* intravenous immunoglobulin, *PLEX* plasmapheresis, *TVP* transvenous pacemaker, *PPM* permanent pacemaker, *WBC count* white blood cell count. Time periods relate to days since first dose of ICI

^*^A – atrial lead complication, V – ventricular lead complication

^**^CHB present on admission

^***^Troponin I was raised, TnT not performed

**Table 2:** Demographic characteristics and co-morbidities of people with complete heart block

Patient Characteristics	*n* (%) (*N* = 7)	*p*-value*
Sex (male)	3 (42.86%)	0.143
Age	*(median79)*	0.241
Medical comorbidities		
Diabetes	1 (14.29%)	1.00
Hypertension	6 (85.71%)	1.00
Coronary artery disease	1 (14.29%)	1.00
COPD	1 (14.29%)	1.00
Obstructive sleep apnea	2 (28.57%)	1.00
Stroke or TIA	1 (14.29%)	1.00
Prior or current smoker	3 (42.86%)	1.00
Prior pericarditis	0 (0%)	
Heart failure	0 (0%)	
Atrial fibrillation	1 (14.29%)	0.286
Hyperlipidemia	5 (71.43%)	1.00
Medications		
Antihypertensive	4 (57.14%)	
Antiplatelet	2 (28.57%)	
Anticoagulant	3 (42.86%)	
Atrioventricular node blocking agent	1 (14.29%)	
Cancer primary		
Lung adenocarcinoma	2 (28.57%)	
Renal cell carcinoma	1(14.29%)	
Vaginal melanoma	1(14.29%)	
Bladder urothelial squamous carcinoma	1(14.29%)	
Renal clear cell carcinoma	1(14.29%)	
Testicular sertoli cell carcinoma	1 (14.29%)	
Stage at initiation of ICI therapy		
Stage IIIb	1 (14.29%)	
Stage IV	6 (85.71%)	
Systemic anti cancer treatments		
Durvalumab	1 (14.29%)	
Intravesical mitomycin C	1 (14.29%)	
Carboplatin & Paclitaxal	1 (14.29%)	
Carboplatin & Pemetrexed	1 (14.29%)	
Durvalumab	1 (14.29%)	
Axitinib	1 (14.29%)	
Cabozantanib	1 (14.29%)	
Radiation therapy	4 (57.14%)	

^*^Association with mortality risk at 180 days (Fisher’s exact test)

**Table 3:** Association between diagnostic tests and survival time and 180 day mortality risk among people with ICI myocarditis who develop CHB

**Diagnostic test**	**Association with survival**		*P*-value for mortality risk*	***N* = **
	**Spearman’s rho**	***p*-value**	**(ranksum)**	
**Laboratory tests**				
White blood cell count	-0.775	0.041	0.245	7
Troponin T	-0.943	0.0048	0.143	6
NT pro-BNP	0.3	0.624	0.157	3
Creatine Kinase	0	1.000	0.480	5
AST	-0.306	0.504	0.699	5
ALT	-0.487	0.268	0.699	7
Albumin	-0.638	0.173	0.766	6
**ECG at admission**				
Ventricular rate	0.812	0.0499	0.245	6
PR interval	0.290	0.637	0.643	5
QRS duration	-0.633	0.128	0.121	7
QT interval	0.479	0.277	0.241	7
QTc interval	-0.316	0.490	0.439	7
Sokolow Lyon Index	-0.655	0.158	0.143	6
Treatment			**Exact p-value**	
IVIG			0.858 *	7
Temporary pacemaker			0.130 *	7
Permanent pacemaker			0.051*	7

^*^Fisher’s exact test

There was a trend towards association between use of a permanent pacemaker and survival (Tables 1 and 3), however this was not statistically significant (*p* = 0.051). Two patients declined permanent pacemaker (PPM) insertion and died shortly thereafter. Five patients underwent PPM insertion, one of whom received a leadless pacemaker. Device lead complications were present in 3 of 4 patients. One patient developed bradycardic arrest secondary to ventricular lead failure to capture (exit block), however was successfully resuscitated and the lead was replaced, after which device function appeared normal. One patient had atrial under-sensing and atrial sensing was disabled. Another patient developed atrial lead dislodgement immediately following the procedure. These two patients were transitioned to ventricular pacing, but did not develop apparent long-term structural sequelae of lead malfunction. No patient underwent magnetic resonance imaging (MRI) following pacemaker insertion.

### All-cause mortality in ICI myocarditis

Several ECG characteristics were strongly associated with reduced mortality, including SLI < 13 mV (*p* = 0.01) and QRS duration > 120 ms (*p* = 0.04). TnT was also strongly associated with increased mortality rate (*p* = 0.01) (Table 3).

### Predicting development of CHB

Baseline ECG characteristics, include first degree AV block and low SLI, were strongly associated with development of CHB (Table 4). All patients who developed CHB had QRS > 120 ms (*p* = 0.003). There was no significant association between laboratory findings and CHB development, however there was a non-significant trend towards increasing incidence of CHB with increasing AST level (OR for 1-unit increase 1.006, *p* = 0.058).

**Table 4:** Association between clinical risk factors and complete heart block among patients with ICI myocarditis using unadjusted logistic regression

	*N* =	OR (95% CI)	*p*-value**
**Laboratory results**			
Troponin T	33	1.00 (1.00 to 1.00)	0.555
Creatine kinase	24	1.00 (1.00 to 1.00)	0.165
WBC count	34	1.143 (0.93 to 1.40)	0.203
AST	30	1.006 (1.0 to 1.01)	0.058
ALT	30	1.005 (0.998 to 1.01)	0.190
NT pro-BNP	17	0.998 (0.99 to 1.00)	0.362
**ECG findings**			
Ventricular rate	34	0.964 (0.92 to 1.01)	0.107
First degree AV block * (PR interval > 200 ms)	28	10.0 (1.15 to 86.88)	0.037
QRS duration * ≥ 130 ms	24	26.4 (2.57 to 271.09)	0.006
QT interval	34	1.009 (0.997 to 1.02)	0.152
QTc interval	34	0.997 (0.98 to 1.02)	0.791
Sokolow Lyon index	33	0.758 (0.60 to 0.96)	0.023
Dual ICI treatment	34	3.3 (0.554 to 19.65)	0.190
Ejection fraction on TTE	29	1.109 (0.995 to 1.24)	0.060

*TTE* transthoracic echocardiogram, *AV* atrioventricular, *AST* aspartate aminotransferase

Unless otherwise indicated with *, investigations are treated as continuous variables. **Wald test

### Cardiac imaging features

There was little evidence of association between TTE assessment of left ventricle systolic function and regional wall motion abnormalities (Table 5). TTE was performed in 31 cases. Twenty-four TTEs were included in analysis of strain imaging. Of the 7 patients with TTE following ICI administration where strain was not performed, one was permanently paced, one TTE was of insufficient quality, and the other 5 TTEs were not processed due to technical issues. There was no strong rank association between global or segmental longitudinal strain and CHB or mortality rates (Table 6). The median values of global, mid and basal longitudinal strain were significantly greater than -18% (> -18% defined as normal). The basal, mid and global strain values were significantly lower than the apical median strain value.

**Table 5:** Sensitivity, hazard ratio for association between positive test and mortality rate, and hazard ratio for mortality among those not tested compared to those tested, among patients with immune checkpoint inhibitor myocarditis, using cox bivariable regression

Diagnostic test	*n* = (34)	Sensitivity	HR for mortality rate (95% CI)	*p*-value	HR for mortality (tested vs not tested) (95% CI)	*p*-value
5^th^ gen Troponin T*	33	97%	6.25 (1.54 to 25.27)	0.01		
Anti-striated antibody	7	57.14%				
QRS duration > 120 ms	34	47%	5.13 (1.06 to 24.78)	0.04		
Sokolow-Lyon Index < 13 mV	33	39.39%	7.42 (1.53 to 35.94)	0.01		
2-D echocardiogram						
LVEF < 50%	29	34.48%	0.22 (0.03 to 1.77)	0.15	1.28 (0.16 to 10.22)	0.82
RWMA	33	33.33%	0.21 (0.03 to 1.70)	0.15		
CMR					16.15 (2.01 to 129.75)	< 0.01
**Diagnostic certainty**	***n* = (%)**		**Mortality rate (deaths per 100 days)**		**HR for mortality (95% CI)**	**p-value**
Definite myocarditis	14 (41.18%)		0.04 (0.01 to 0.29)		1.00	
Probable myocarditis	7 (20.59%)		0.09 (0.01 to 0.61)		2.10 (0.13 to 33.58)	0.13
Possible myocarditis	13 (38.24%)		0.47 (0.22 to 0.98)		10.67 (1.31 to 87.09)	0.03
Test for departure from linear trend with increasing diagnostic certainty: LR χ^2^ = 0.16, *p* = 0.69, 1 d.f						

^*^HR given for TnT > 1000 ng/L

**Table 6:** Association between strain imaging, outcome variables and predictor variables among patients with immune checkpoint inhibitor myocarditis

**Level of longitudinal strain**								
**Sensitivity (> -18%) for ICI myocarditis**	**Global Strain**		**Apical Strain**		**Mid strain**		**Basal strain**	
	83.33%		58.33%		91.67%		87.5%	
**Comparison**								
**(Sign rank null hypothesis)**	**Median**	***P* = **	**Median**	***P* = **	**Median**	***P* = **	**Median**	***P* = **
Strain = -18%	-15.56%	< 0.001	-16.79%	0.188	-15.07%	< 0.001	-14.78%	0.001
Strain = apical strain		0.007		–-		0.010		0.043
**Outcome variables**								
**(Cox regression)**	**HR (95% CI)**		**HR (95% CI)**		**HR (95% CI)**		**HR (95% CI)**	
CHB	0.864 (0.67 to 1.11)	0.250	0.902 (0.75 to 1.09)	0.283	0.895 (0.69 to 1.16)	0.393	0.936 (0.78 to 1.13)	0.491
Death	1.027 (0.80 to 1.32)	0.837	1.016 (0.84 to 1.23)	0.874	0.969 (0.76 to 1.23)	0.798	0.979 (0.81 to 1.19)	0.827
**Predictor variables**	**Correlation**		**Correlation**		**Correlation**		**Correlation**	
Troponin T	Rho = -0.432	0.040	Rho = -0.145	0.508	Rho = -0.483	0.020	Rho = -0.524	0.010
PR interval	Rho = -0.401	0.080	Rho = -0.199	0.401	Rho = -0.381	0.097	*r* = -0.322 *	0.167
QRS duration	Rho = -0.056	0.796	Rho = 0.180	0.399	Rho = -0.185	0.388	Rho = -0.085	0.695
Sokolow Lyon Index	*r* = 0.4402 *	0.036	Rho = 0.102	0.644	Rho = -0.011	0.962	*r* = 0.630 *	0.001

*CHB* complete heart block, *CI* confidence interval

^*^Linear regression used in place of Spearman correlation based on a review of scatter plots, Pearson correlation coefficient presented

^**^Sign rank null hypothesis

CMR was associated with modest sensitivity of 65% for diagnosis of ICI myocarditis; however, it was not prognostically significant and there was suggestion of selection bias (Table 5). CMR evidence of myocarditis (definite myocarditis) was available for 41.18% (14/34) of cases. There was no association between mortality and CMR results (Exact *p* = 0.35). Lack of CMR during or after index hospitalization was associated with a 16-fold increase in mortality rate at 180 days (HR for lack of CMR 16.15, 95% CI 2.01 to 129.75, *p* < 0.01). Failure or inability to undergo completed CMR was strongly associated with CHB incidence (Exact *p* = 0.012). There was a strong linear trend towards increasing mortality with increasing diagnostic certainty (Wald test *p*-value 0.016 for difference in HR, *p* = 0.69 for test for departure from linear trend, see Table 5).

### Myocarditis prognostic approach

**Fig. 2:**
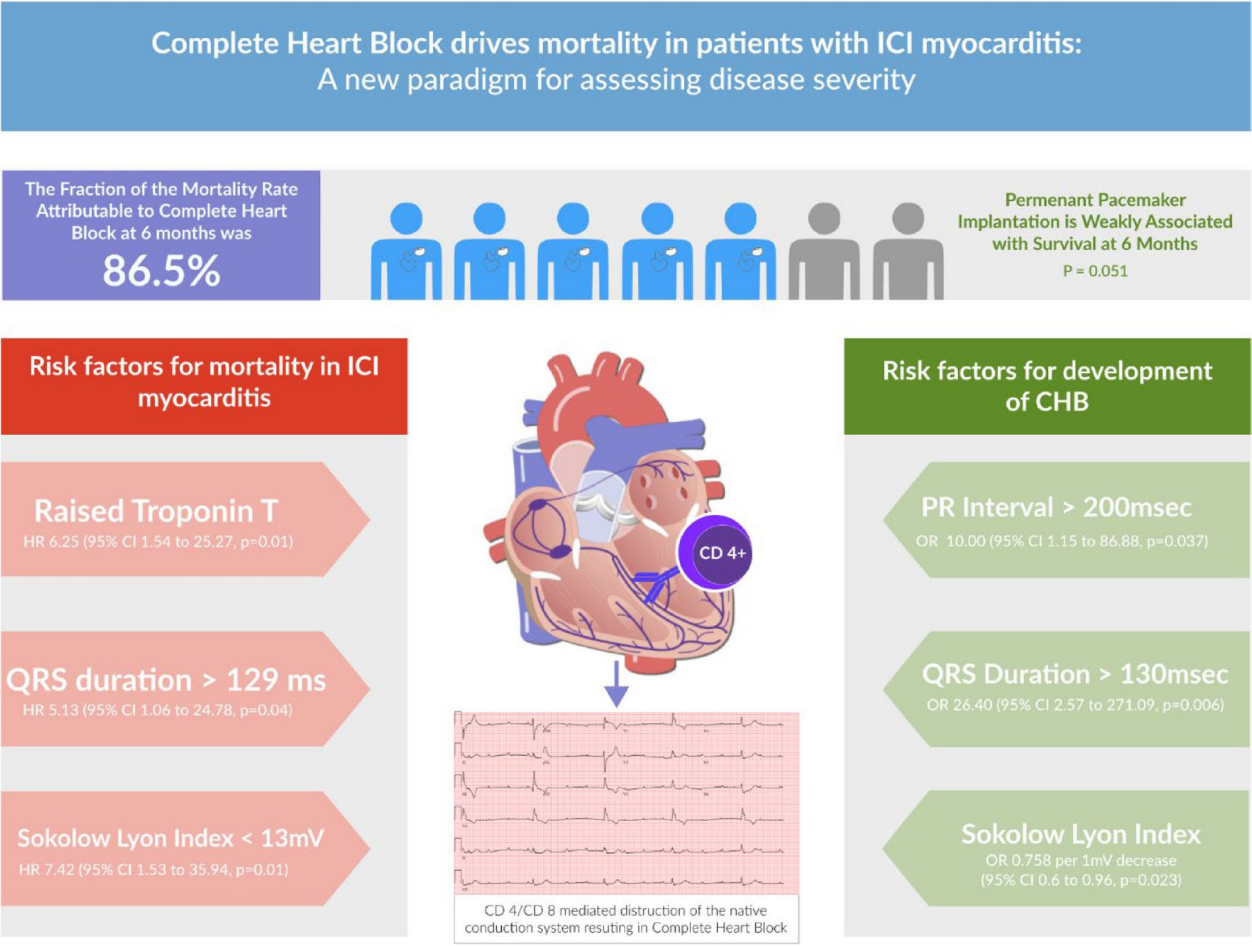
Central Illustration

Taking into account parameters identified as being associated with increased mortality, a prognostication scale of ICI myocarditis was proposed based on the presence of the following prognostic characteristics during admission for ICI myocarditis: 1) Troponin T > 1000 ng/dL, 2) ECG PR interval > 200 ms, 3) ECG QRS interval > 120 ms, and 4) ECG Sokolow Lyon Index > 14 mV. Patients without any of these characteristics were classified as mild ICI myocarditis, those with one or more characteristics as having moderate ICI myocarditis, and those with complete heart block as having severe myocarditis. There were no deaths in the group with mild myocarditis (Table 7). For each increase in severity grade, there was a sixfold increase in mortality rate, and this difference was statistically significant (*p* < 0.001) (Fig. 2).

**Table 7:** Severity classification for immune checkpoint inhibitor myocarditis based on risk of developing CHB

**Predictor variables**		Mild All of:	Moderate Any 1 of:	Severe
Troponin T		≤ 1000 ng/dL	TnT > 1000 ng/dL	-
ECG*				
PR interval		≤ 200 ms	> 200 ms	-
QRS duration		≤ 120 ms	> 120 ms	-
Sokolow Lyon Index		> 14 mV	≤ 14 mV	-
CHB		Not present	Not present	Present
**Mortality rate (100 person-days) (95% CI)**		0	0.126 (0.05 to 0.33)	0.741 (0.31 to 1.78)
**Proportion (%)**		7 (20.59%)	20 (58.82%)	7 (20.59%)
**Cox HR for linear association between severity and mortality rate**			HR 6.273 (2.127 to 18.506), *p* < 0.001	

*ECG electrocardiogram refers to admission ECG

## Discussion

Diagnosis and prognostication in ICI myocarditis is a significant challenge. This study demonstrates the clinically important finding that the development of CHB is strongly associated with early mortality in ICI myocarditis. At 6 months, 86.5% of all-cause mortality was attributed to CHB. The remaining deaths occurred almost exclusively in patients with risk factors for development of CHB, such as prolonged QRS and reduced SLI. These findings are consistent with the initial case series of ICI myocarditis, which demonstrated infiltration of myocardium and conduction system, including the sinoatrial and atrioventricular nodes, with T-cells and macrophages [2].

This large study reviewing of over 8,500 patients receiving ICI across three states in a tertiary referral medical institution, lends additional perspective into the overall low incidence of ICI myocarditis, and insight into the predominantly conduction related mechanism for mortality. There is a range of previously reported incidences of CHB in the literature, with incidence rates of 42% (15/36 cases) in Cautela et al., [11], 15.2% (19/125) in Power et al., [7], and 8.6% (3/35) cases identified in Mahmood et al. [12]; this comes to a cumulative prevalence of 18.88% (37/196 cases) which is similar to our study’s estimate of 20.58%. The total effect of CHB on mortality however appears higher, likely due to the use of survival time analysis. This is due to this study’s unique cohort design, using mortality rate rather than risk to estimate attributable fraction and hazard ratio. Mortality risk is comparable to other ICI myocarditis cohorts, indicating good external validity [2–4].

Through this cohort study, we illustrate a framework for prognostication in ICI myocarditis which is dependent on more readily available troponin and ECG data, with a focus on prediction of CHB incidence. This framework is in keeping with existing evidence that both troponin and ECG measurements have significant prognostic value in ICI myocarditis [4, 7, 11, 13, 14]. In a condition where more advanced diagnostics are difficult to obtain, and where prognostic value of other investigations is limited, a focus on arrhythmogenic complications of this condition can help determine the appropriate level of care for these critically ill individuals.

Myocardial strain has been proposed as an early marker for ICI myocarditis, and is associated with elevated troponin levels [15]. It has also been suggested as a predictor of mortality [16, 17]. Although our numbers are small in the present study, we observe that despite high sensitivity, GLS did not have significant prognostic value. There are significant difficulties in obtaining CMR in this cohort in addition to limitations of low sensitivity [8, 9, 13]. There was potential bias in results of CMR testing, as CMR and echo strain data were frequently not performed in assessment of ICI myocarditis. Failure or inability to complete these studies was strongly associated with high mortality rates. The most recent guidelines relating to ICI myocarditis have removed CMR from diagnostic criteria, likely due to limited sensitivity [13, 14]. Studies examining CMR in ICI myocarditis rely on patient registries for data [8, 9]. This study demonstrates that such patients who have a CMR are much less likely to die from myocarditis. They therefore may have different (and less severe) disease on imaging than those who cannot tolerate the procedure, resulting in selection bias.

Further research including a focus on prevention and management of CHB in ICI myocarditis is required. Research to date has focused on use of immunosuppression [11]. Despite the small sample size, this study provides some data to support use of permanent pacemakers in treatment of ICI myocarditis with CHB. There may be a role for empiric device insertion in patients with high-risk features for CHB development. Given the high burden of device lead complications, use of leadless devices may be a consideration in this cohort, provided it is in line with overall patient goals of care. Early recognition of patients who are at high risk of progression to this often-fatal arrhythmia should be the focus of initial clinical evaluation.

### Study limitations

There are limitations to this study. ICI myocarditis is a rare condition, and this is reflected in the small study size. This prevents use of multivariate analysis; however statistical power is retained. This is due to the high mortality rate in ICI myocarditis in general and the relatively large difference in mortality rate between those with and without CHB. This paper doesn’t examine cause-specific analysis due to the high number of out-of-hospital deaths in this cohort, as many patients were discharged to hospice care. It is challenging to adjudicate cause of death in a manner which would avoid differential misclassification, so all-cause mortality rate was used. The study cohort was limited to hospitalized patients, so results may not be generalized to the outpatient setting. Information bias was minimized by applying a standardized definition, and use of three physician majority consensus. It is possible that cases were missed where diagnosis of myocarditis was not captured in coding. The approach to severity stratification presented in Table 6 will require external validation. For associations with CHB, there were fewer than 7 cases, which introduces a risk of unstable regression analysis. This doesn’t appear to be the case, as results of the primary association can be verified with non-parametric methods.

## Conclusion

Mortality in ICI myocarditis is strongly associated with CHB. ECG parameters and troponin level may help identify those who may be higher risk of development of CHB.

## Data Availability

Irrevocably anonymized is available upon reasonable request via email to osheam12@tcd.ie.

## Abbreviations

ICI: Immune checkpoint inhibitors
CHB: Complete heart block
TnT: 5^Th^ generation troponin T
ECG: Electrocardiogram
CMR: Cardiac magnetic resonance imaging
TTE: Transthoracic echocardiogram
SLI: Sokolow Lyon Index
